# Achievement goals and life satisfaction: the mediating role of perception of successful agency and the moderating role of emotion reappraisal

**DOI:** 10.1186/s41155-017-0078-4

**Published:** 2017-12-22

**Authors:** Wangshuai Wang, Jie Li, Gong Sun, Zhiming Cheng, Xin-an Zhang

**Affiliations:** 1 Shanghai Jiao Tong University, 1954 Huashan Road, Shanghai, China; 2 Shanghai University, 99 Shangda Road, Shanghai, China; 3 Central University of Finance and Economics, 39 South College Road, Beijing, China; 4 Macquarie University, Balaclava Road, Sydney, Australia

**Keywords:** Achievement goals, Perception of successful agency, Emotion reappraisal, Life satisfaction

## Abstract

Achievement goals are cognitive representations that guide behavior to a competence-related future end state. Existing theories and empirical findings suggest that achievement goals are potentially related to life satisfaction. However, the relationship between achievement goals and life satisfaction remains relatively unexplored in the psychology literature. In this study, we examined how, why, and when achievement goals affect life satisfaction using original survey data from China. The results suggest that achievement goals were positively related to life satisfaction (*R*^2^ = .20, 90% CI [.11, .26]), that the perception of successful agency fully mediated the relationship between achievement goals and life satisfaction (*R*^2^ = .22, 90% CI [.12, .27]), and that emotion reappraisal moderated the relationship between achievement goals and life satisfaction (*R*^2^ = .34, 90% CI [.23, .39]). Our study indicates that achievement goals have a positive influence on life satisfaction and help to elucidate the mechanism and boundary condition of this influence.

## Background

An achievement goal refers to “a future-focused cognitive representation that guides behavior to a competence-related end state that the individual is committed to either approach or avoid” (Hulleman, Schrager, Bodmann, & Harackiewicz, 2010, p. 423). In the past three decades, there has been a large body of literature published on achievement goals (see Hulleman et al., 2010, for a meta-analytic review). Existing research shows that individuals differ in their behaviors and preferences in pursuit of achievement goals (Harackiewicz & Sansone, 1991). For example, one may easily recall that in school years, certain students worked hard and performed well on exams, demonstrating high achievement goals. In contrast, other students were not strongly concerned regarding academic performance, did not study, and had poor performance in exams, which denoted low motivation for achievement goals.

One stream of research has identified the antecedents of achievement goals. For example, age is negatively related to achievement goals; females have a stronger mastery of goal orientation than males in an academic setting, whereas self-efficacy and perceived social environment, including peer relationships and sense of belonging, are positive predictors of achievement goals (Ablard & Lipschultz, 1998; Anderman & Anderman, 1999; Bong, 2009; Phillips & Gully, 1997).

More recently, attention has been directed to the consequences of pursuing achievement goals. For instance, achievement goals positively predict long-term academic performance (Harackiewicz, Barron, Tauer, Carter, & Elliot, 2000). Moreover, achievement goals can activate intrinsic motivation (Cury, Elliot, Sarrazin, Da Fonseca, & Rufo, 2002). Based on this finding, Lee, Sheldon, and Turban (2003) argue that achievement goals promote academic enjoyment. In contrast, researchers also find that negative emotions can be exacerbated by achievement goals due to high expectations. For example, students aspiring for high achievement goals may experience more anxiety during tests (Flanagan, Putwain, & Caltabiano, 2015).

The existing literature on life satisfaction shows that demographic variables, including gender, age, income, and education level, are associated with life satisfaction (Gannon & Ranzijn, 2005; Johnson & Krueger, 2006) and that a person who is more satisfied with life is more diligent, performs better at his/her job, and has a higher commitment to the organization (Efraty, Sirgy, & Claiborne, 1991; Greenhaus, Bedeian, & Mossholder, 1987). More recent research finds that expectation and aspiration are important to job and life satisfaction (Cheng, Wang, & Smyth, 2014; Gao & Smyth, 2010). Similarly, academic goal progress is found to influence both academic and life satisfaction (Ojeda, Flores, & Navarro, 2011; Singley, Lent, & Sheu, 2010). Furthermore, Keller and Siegrist (2010) suggest that both goal pursuit and life satisfaction are psychological resources.

Although these aforementioned studies suggest potential connections between achievement goals and life satisfaction, few studies have directly tested this relationship. In particular, it is unclear in the literature whether achievement goals influence life satisfaction in a positive or a negative way. On the one hand, individuals with high achievement goals can be substantially motivated by mental energy in the face of challenge (Grant & Dweck, 2003). On the other hand, these people also need to make a concerted effort in the stressful and laborious process of pursuing their goals (Senko & Harackiewicz, 2005).

People are paying increasing attention to the improvement of the quality of life. Life satisfaction’s fundamental role and indispensability have been acknowledged by worldwide respondents (Diener, Oishi, & Lucas, 2003). Therefore, to help fill the gaps in the literature and to respond to the practical necessity, this research examines the association between achievement goals and life satisfaction. We also investigate why and when achievement goals influence life satisfaction by examining the underlying mechanism through perception of successful agency and the boundary condition of emotion reappraisal. It is also surprising that little research on achievement goals, successful agency, and emotional reappraisal have been conducted in non-Western cultures (e.g., Chinese culture), which leaves a potentially rewarding empirical research area to be explored. Existing studies suggest that there are significant cultural differences in positive psychology (e.g., Diener, Diener, & Diener, 1995; Spencer-Rodgers, Peng, Wang, & Hou, 2004). It is, therefore, very important to examine these constructs using data drawn from non-Western cultures.

Taken together, in this research, we first answer an important but unresolved question: what is the relationship between achievement goals and life satisfaction? We further advance our study by testing the potential mediation and moderation of this relationship. The current research also has significant practical implications for the general public—including but not limited to workers and students—on the means to successfully pursue greater happiness.

Life satisfaction is a global cognitive judgment across a broad set of activities concerning one’s quality of life (Diener et al., 2003; Matud, Bethencourt, & Ibáñez, 2014). Various factors are related to life satisfaction, such as finances (Johnson & Krueger, 2006), family and marital relationships (Adams, King, & King, 1996; Cheng & Smyth, in press), health conditions (Canha, Simões, Matos, & Owens, 2016), coping strategies (Nunes, Melo, Júnior, & Eulálio, 2016), and sexual behaviors (Cheng & Smyth, 2015).

Although the direct evidence for the link between achievement goals and life satisfaction is limited, previous research has provided some indirect support. For instance, the self-determination theory theorizes two forms of motivation, which are controlled motivation and autonomous motivation (Ryan & Deci, 2000). Controlled motivation originates either from self-imposed pressures or from external pressures, such as pleasing others or complying with demands, both of which have an externally perceived locus of causality. In contrast, autonomous motivation stems from one’s self, thereby having an internally perceived locus of causality (Weinstein & Ryan, 2010). Setting high achievement goals, in many cases, reflects one’s own values; thus, it is internally driven and inspires autonomous motivation (Cury et al., 2002). Importantly, literature based on self-determination theory indicates that autonomous motivation positively contributes to well-being (Ryan & Deci, 2000).

Moreover, individuals often want to maintain a sense of control, expecting everything to be in line with their plans (Park & Baumeister, 2017). However, there are always discrepancies between expectations and reality. Under certain circumstances, the experiences of hardships often demotivate people and make them feel dissatisfied with life. Achievement goals can provide a person with motivation (Pintrich, 2000), which serves as mental energy helpful in overcoming the difficulties and obstacles in life (Capa, Audiffren, & Ragot, 2008). As a result, people who set achievement goals for themselves are less affected by experiences that can have negative effects on life satisfaction.

Furthermore, researchers find that setting achievement goals is helpful to one’s educational and occupational performance, since it results in better grades at school and upward career mobility (Gould, 1980; Harackiewicz et al., 2000; Harackiewicz, Barron, Tauer, & Elliot, 2002). The successes in academic and job domains boost self-efficacy and self-esteem (Bachman & O’Malley, 1977; Leary, Tambor, Terdal, & Downs, 1995; Tay, Ang, & Van Dyne, 2006), both of which can enhance satisfaction with life (Du, Bernardo, & Yeung, 2015; Joseph, Royse, Benitez, & Pekmezi, 2014). Therefore, we propose the following hypothesis:

*Hypothesis 1: Achievement goals are positively correlated with life satisfaction.*

Perception of successful agency is a sense of determination to be successful in pursuing goals, by which hope is fueled (Snyder et al., 1991). Perception of successful agency is conceptually similar to self-efficacy, and they are shown to be positively and moderately correlated (Magaletta & Oliver, 1999). However, successful agency is more future-oriented than is self-efficacy (Snyder et al., 1991). Thus, perception of successful agency is more closely related to achievement goals compared to self-efficacy.

We hypothesize that achievement goals are positively related to perception of successful agency. This is because achievement goals usually lead people to maintain high standards and strive to accomplish difficult tasks (Phillips & Gully, 1997). After making every effort to ensure success, people are likely to hold positive expectations towards the outcomes. This notion is supported by the effort justification theory (Aronson & Mills, 1959), which states that people’s expectations are in direct proportion to his/her effort. As expectations continue rising, they tend to attribute an even greater value to an outcome that they put effort into achieving.

In addition, we propose that perception of successful agency is positively associated with life satisfaction for two reasons. First, perception of successful agency makes one’s life meaningful. Feldman and Snyder (2005) suggest that perception of successful agency per se is actually a component of meaning, because factor analysis shows a single factor underlying the two constructs. People who feel that their life is more meaningful also report higher satisfaction with life (Park, Park, & Peterson, 2010; Steger, Frazier, Oishi, & Kaler, 2006). Second, according to the notion that hope copes with obstacles and enhances meaning in life, several empirical research has revealed a positive relationship between hope and life satisfaction (Bailey, Eng, Frisch, & Snyder, 2007; Bronk, Hill, Lapsley, Talib, & Finch, 2009; O’Sullivan, 2011; Przepiorka, 2017). Because perception of successful agency is one dimension of hope, we expect its relationship with life satisfaction to be similar. Based on the above discussion, we hypothesize that:

*Hypothesis 2: Perception of successful agency mediates the relationship between achievement goals and life satisfaction.*

Individuals exert considerable control over their emotions but differ in their use of specific emotion regulation strategies. Of these, the two most widely used strategies are reappraisal and suppression (Gross & John, 2003). Emotion reappraisal is a cognitive change of emotional impact by construing a potentially emotion-eliciting situation. For example, people can feel upset or frustrated in a traffic jam. However, if drivers reevaluate the current situation and consider a traffic jam as an unexpected opportunity to enjoy the beautiful scenery along the road, they can probably feel better off. This act of recognizing and changing the pattern of thoughts falls into emotion reappraisal. Compared with suppression, reappraisal is a much more effective regulation strategy (Gross, 1998; Gross & John, 2003). People who habitually use emotion reappraisal are less likely to be depressed (Feinberg, Willer, Antonenko, & John, 2012), experience more positive emotions and fewer negative emotions, and have better social functioning (Gross & John, 2003).

Achievement goals promote one’s expectation of the end state, which cannot always remain perfect. Failing to meet a goal means that most of the early efforts become sunk costs, which leads to decreased self-confidence and increased self-blame. These negative self-cognitions, in turn, trigger severe emotional reactions (Brown & Dutton, 1995), such as depression and anxiety (Ellenhorn, 2005; Hewitt & Flett, 1991). Consequently, when emotion reappraisal is low, the negative consequences caused by failure are unable to be adjusted in time, which lowers a person’s perceived quality of life. In this condition, the positive relationship between achievement goals and life satisfaction is attenuated. In contrast, when emotion reappraisal is high, individuals take an optimistic attitude to negotiate stressful situations and thus become more immune to the pressure of goal failure (Gross & John, 2003). As a result, their satisfaction with life remains positively correlated with achievement goals. Therefore, we propose the following hypothesis:

*Hypothesis 3: Emotion reappraisal moderates the positive relationship between achievement goals and life satisfaction, such that the relationship is stronger when emotion reappraisal is high rather than low.*

## Methods

### Participants and procedures

Data were collected via a survey from a sample of 225 participants in mainland China in late 2016 using Sojump (http://www.sojump.com), which is a professional online survey platform similar to Amazon’s Mechanical Turk. Sojump has a large, diverse workforce consisting of over 2.6 million users with different demographic backgrounds. It provides reliable crowdsourcing services and has been used in previous psychological research (e.g., Chen, Austin, Miller, & Piercy, 2015; Li, Chen, & Huang, 2015). Respondents in the current study were randomly recruited from Sojump. Before starting the survey, they were told that their responses would remain confidential. After completing the survey, they received a monetary reward. Previous research has documented that giving a monetary reward to participants can improve their motivation in responding, thus being beneficial to the quality of survey data (Esterman, Reagan, Liu, Turner, & DeGutis, 2014). Online studies even amplify this advantage. A monetary incentive can inspire participants to respond carefully when researchers are unable to monitor how the participants fill in the survey, which is why plenty of psychological studies using online platforms pay for participation (e.g., Saleem, Anderson, & Barlett, 2015; Stroessner, Scholer, Marx, & Weisz, 2015).

All of the respondents were adults. Among the respondents, 106 were males, and 119 were females; 73, 23, and 4% of them were 18–35, 36–53, and above 54 years old, respectively. Forty-one and 42% of the respondents’ monthly salary ranged from 2000 to 4000 yuan and from 4001 to 6000 yuan, respectively. The majority of the sample was well-educated: 53, 21, and 6% of them held bachelor’s degrees, master’s degrees, and PhDs as their highest degrees, respectively. With regard to job tenure, 63% of the participants had worked in their companies for more than 4 years, whereas 29 and 8% of them had worked in their companies for 2 to 3 years and less than 2 years, respectively.

### Measures

We created a Chinese version of a set of measures for achievement goals, emotion reappraisal, perception of successful agency, life satisfaction, and social desirability. To ensure the accuracy of the translation, we followed Brislin’s (1986) translation and back-translation procedures. Specifically, the items of the scales were first translated into Chinese by a native Chinese speaker with excellent knowledge of English. Next, this process was reversed by a native English speaker with excellent command of Chinese. For a very small number of items, the back-translation procedure resulted in inconsistencies. However, these inconsistencies were resolved by discussion between the two translators and the researchers. To further validate the translation, we conducted a pretest involving 20 randomly recruited participants from Sojump before implementing the formal survey. After the completion of the pretest survey, participants declared that the survey questions were easily understood and that there were no barriers to responding. The participants in the pilot study were not included in the final sample because combining two sources of samples may rule in the confounding due to different times of data collection. Moreover, we performed another set of statistical analyses with the participants in both the pilot and formal study. No significant difference was found compared with the current results. Therefore, we only reported the analyses in the formal study.

#### Achievement goals

Achievement goals were measured by the Achievement Goal Striving Scale, which is a ten-item scale adapted from Goldberg’s (1999) International Personality Item Pool (IPIP). It has been widely used in previous studies and has proven to have good reliability and validity (Hirschfeld, Lawson, & Mossholder, 2004). On a seven-point scale (1 = *not at all characteristic*; 7 = *very characteristic*), participants rated how characteristic each statement best described themselves. An example item is “I go straight for the goal.” We used Omega to estimate reliability, because compared to Cronbach’s alpha, Omega provides a better estimate with more appropriate assumptions (Crutzen & Peters, 2017; McNeish, in press). All of the items were averaged to create the score for achievement goals (Omega = .91).

#### Emotion reappraisal

Emotion reappraisal was assessed using the reappraisal subscale of the Emotion Regulation Questionnaire. This instrument is a six-item measure developed by Gross and John (2003). Participants indicated their agreement with each item on a seven-point scale (1 = *strongly disagree*; 7 = *strongly agree*). An example item is “I control my emotions by changing the way I think about the situation I’m in.” All of the items were averaged to create the score for emotion reappraisal (Omega = .96).

#### Perception of successful agency

We measured perception of successful agency using Snyder et al.’s (1991) Agency subscale of the Hope Scale (e.g., Chang, 2003; Gallagher & Lopez, 2009), which consists of four items. Participants were asked to evaluate the extent to which each item applied to them on a seven-point scale (1 = *definitely false*; 7 = *definitely true*). An example item is “I energetically pursue my goals.” All of the items were averaged to create the score for perception of successful agency (Omega = .95).

#### Life satisfaction

We assessed life satisfaction using the five-item Satisfaction with Life Scale developed by Diener, Emmons, Larsen, and Griffin (1985). On a seven-point scale (1 = *strongly disagree*; 7 = *strongly agree*), participants reported the overall satisfaction with their life under different indicators. An example item is “In most ways my life is close to my ideal.” All of the items were averaged to create the score for life satisfaction (Omega = .94).

#### Control variables

In the survey, we also collected information on some important variables that are potentially correlated with life satisfaction, such as gender, age, income, education level (Gannon & Ranzijn, 2005; Johnson & Krueger, 2006), job tenure (Adams et al., 1996), and social desirability bias. We used the Marlowe–Crowne Social Desirability Scale (Form C) with 13 true-false format items (Reynolds, 1982) to assess social desirability (Omega = .77). An example item is “It is sometimes hard for me to go on with my work.”

### Data analysis

We began the analyses by conducting a series of confirmatory factor analyses using LISREL8.8, to verify the distinctness of the variables included in our models. Because our sample size was relatively small, we constructed item parcels in these confirmatory factor analyses. Specifically, four indicators were formed for constructs that contained more than four items by sequentially grouping the highest loading items with the lowest loading ones (Little, Cunningham, Shahar, & Widaman, 2002). After parceling, the total number of indicators decreased to 16, since the number of parcels for each construct was four. We assessed the models by comparing four indicators of fit, including the chi-square/degrees of freedom ratio (*χ*^2^/*df*), comparative fit index (CFI), non-normed fit index (NNFI), and root mean square error of approximation (RMSEA). Good fits are obtained when *χ*^2^/*df* is less than 5 and RMSEA is less than .10, whereas NNFI and CFI are greater than or equal to .90 (Bentler, 1990; Steiger, 1990).

Prior to hypothesis testing, we conducted exploratory factor analyses to ensure that the scales used retained their intended structure (Crutzen & Peters, 2017). Next, correlations among study variables were calculated using Pearson’s correlation coefficients, providing initial support for the hypotheses. Next, we performed hierarchical regressions using SPSS for the purpose of hypothesis testing, in which independent variables were mean-centered to reduce multicollinearity (Cohen, Cohen, West, & Aiken, 2003). Afterwards, as a robustness check for small samples (Preacher & Hayes, 2008), we used a bias-corrected and accelerated bootstrapping procedure (5000 samples were taken) to further examine the achievement goal–perception of successful agency–life satisfaction link. Next, simple slope analysis was applied to probe the nature of the interaction effect (Aiken & West, 1991). Finally, we employed another statistical analysis, which included both successful agency and emotion reappraisal in a single model. Again, we adopted the bootstrapping method as in Model 5 in Hayes (2013). As suggested by Cohen (1990), we reported all effect sizes and confidence intervals in the statistical analyses. Fisher’s *z* and its 95% confidence intervals were calculated in the correlational analysis (Rosenthal, 1991). We chose *R*^2^ as the index of effect sizes for regression analyses and computed the 90% confidence interval for each *R*^2^ (Smithson, 2001).

To support disclosure and replication in scientific research (Peters, Abraham, & Crutzen, 2015) and facilitate future meta-analyses, the data, syntax and statistical outputs used in the present study are available at https://pan.baidu.com/s/1qXLFvq8.

## Results

### Exploratory factor analyses

The measurement instruments were in line with their intended structure, as a single latent variable was observed for each construct, and all scales used in this research were unidimensional (Crutzen & Peters, 2017).

### Measurement model results

The baseline model contained four factors: achievement goals, emotion reappraisal, perception of successful agency, and life satisfaction. We also examined six alternative models against the baseline model. As shown in Table 1, the results suggested that the baseline model fits the data reasonably well (*χ*^2^ (98) = 348.79, CFI = .96, NNFI = .95, RMSEA = .09). The alternative models all exhibited significantly poorer fit than the baseline model. Therefore, we treated the four variables as distinct constructs in later analyses.

**Table 1:** Comparison of results from the measurement models

Model	Description	*χ* ^2^	*df*	Δ*χ*^2^	CFI	NNFI	RMSEA
Null model	All the indicators are independent	7781.41	114		.01	− .05	.54
Baseline model	Four factors	348.79	98		.96	.95	.09
Model 1	Three factors: achievement goals and perception of successful agency were combined into one factor	1078.99	101	730.20**	.87	.84	.20
Model 2	Three factors: achievement goals and life satisfaction were combined into one factor	1360.17	101	1011.38**	.83	.80	.23
Model 3	Three factors: achievement goals and emotion reappraisal were combined into one factor	1453.42	101	1104.63**	.82	.79	.24
Model 4	Three factors: perception of successful agency and life satisfaction were combined into one factor	1131.96	101	783.17**	.86	.84	.21
Model 5	Three factors: perception of successful agency and emotion reappraisal were combined into one factor	1434.94	101	1086.15**	.82	.79	.24
Model 6	Three factors: emotion reappraisal and life satisfaction were combined into one factor	1409.60	101	1060.81**	.82	.79	.24

*N* = 225; Δ*χ*^2^ is the change of *χ*^2^ compared with the baseline model

**p < .01

### Descriptive statistics and correlations

All scales met the distributional assumptions with skewness and kurtosis values lower than ± 1. More specifically, the absolute values for skewness (kurtosis) ranged from .02 to .59 (.19 to .69). The descriptive statistics and correlations among variables are presented in Table 2. Age was positively related to gender (*r* = .16, *p* < .05; Fisher’s *z* = .16, 95% CI [.03, .29]) and income (*r* = .30, *p* < .01; Fisher’s *z* = .31, 95% CI [.18, .44]); education was positively correlated with income (*r* = .24, *p* < .01; Fisher’s *z* = .24, 95% CI [.11, .37]) and negatively correlated with job tenure (*r* = − .26, *p* < .01; Fisher’s *z* = − .27, 95% CI [− .14, − .40]). Consistent with our hypotheses, achievement goals had a significant positive correlation with life satisfaction (*r* = .42, *p* < .01; Fisher’s *z* = .45, 95% CI [.32, .58]) and perception of successful agency (*r* = .83, *p* < .01; Fisher’s *z* = 1.12, 95% CI [.99, 1.25]). Perception of successful agency was also significantly related to life satisfaction (*r* = .44, *p* < .01; Fisher’s *z* = .47, 95% CI [.34, .60]).

**Table 2:** Descriptive statistics, coefficient alphas, and zero-order correlations between variables

	*M*	SD	1	2	3	4	5	6	7	8	9	10
1. Achievement goals	5.08	1.42	(.90)									
2. Emotion reappraisal	4.27	1.67	.35**	(.95)								
3. Perception of successful agency	5.38	1.38	.83**	.27**	(.95)							
4. Life satisfaction	4.55	1.42	.42**	.36**	.44**	(.93)						
5. Gender			− .17*	− .04	− .07	− .02						
6. Age	2.25	.50	.03	− .10	.01	.01	.16*					
7. Income	2.76	1.14	.12	− .05	.15*	.10	− .12	.30**				
8. Education	3.44	.76	− .05	− .06	.01	− .05	− .05	.12	.24**			
9. Job tenure	2.70	.97	− .05	− .02	.01	.10	.02	.06	− .02	− .26**		
10. Social desirability	1.52	.31	.12	.43**	.09	.12	.02	− .05	− .07	− .18**	.05	(.77)

*N* = 225; age: 1 = less than 18 years old, 2 = 18–35 years old, 3 = 36–53 years old, 4 = more than 54 years old; (monthly) income: 1= less than 2000 yuan; 2 = 2000–4000 yuan; 3 = 4001–6000 yuan; 4 = more than 6000 yuan; education: 1= high school, 2 = some college, 3 = bachelor’s degree, 4 = master’s degree, 5 = doctoral degree; job tenure: 1 = less than 2 years, 2 = 2–3 years, 3 = 4–5 years, 4 = more than 6 years; social desirability: 1 = true, 2 = false

^*^p < .05

^**^p < .01

### Hypotheses test results

Table 3 displays the results of the regression analyses for testing Hypothesis 1 (achievement goals are positively related to life satisfaction) and Hypothesis 2 (perception of successful agency mediates the relationship between achievement goals and life satisfaction). The results supported these hypotheses. First, achievement goals were positively and significantly related to life satisfaction (*β* = .42, *p* < .01; *R*^2^ = .20, 90% CI [.11, .26]), supporting Hypothesis 1. Second, to test mediation, we followed Baron and Kenny’s procedure (1986).

**Table 3:** Results for main effect and mediation from hierarchical regression

	Model 1: Perception of successful agency	Model 2: Life satisfaction	Model 3: Life satisfaction
Controls			
Gender	.08	.06	.04
Age	.05	− .04	− .03
Income	.07	.07	.06
Education	.06	.01	− .01
Job tenure	− .05	.12	.10
Social desirability	.01	.07	.07
Main effects			
Achievement goals	.85**	.42**	.20
Perception of successful agency			.25*
*F*	77.19**	7.94**	7.71**
*R* ^2^	.71	.20	.22
Adjusted *R*^2^	.70	.18	.19
Δ*R*^2^			.02**
90% CI	[.65, .74]	[.11, .26]	[.12, .27]

CIs are confidence intervals for effect sizes; *N* = 225

^*^p < .05

^**^p < .01

In Model 1, we regressed successful agency on the control variables and achievement goals. In Model 2, we regressed life satisfaction on the same variables as in Model 1. In Model 3, we regressed life satisfaction on the controls, achievement goals, and successful agency. The results supported Hypothesis 2. First, achievement goals were significantly related to successful agency (*β* = .85, *p* < .01; *R*^2^ = .71, 90% CI [.65, .74]). Second, achievement goals were significantly related to life satisfaction (*β* = .42, *p* < .01; *R*^2^ = .20, 90% CI [.11, .26]). Third, successful agency was significantly related to life satisfaction (*β* = .25, *p* < .01; *R*^2^ = .22, 90% CI [.12, .27]), even after achievement goals were controlled for. In addition, the insignificant coefficient for achievement goals (*β* = .20, *p* > .05) indicated that successful agency completely mediated the relationship between achievement goals and life satisfaction. Furthermore, a 5000 resample bootstrap suggested a significant indirect effect via successful agency (*b* = .24, SE = .09, 95% CI [.06, .42]). This finding again supported Hypothesis 2. Additionally, following MacKinnon, Lockwood, Hoffman, West, and Sheets (2002), we calculated the *z* coefficient, which results from the division of the mediated effect by its standard error. Consistent with prior findings, the calculation yielded a significant result (*z* value = 2.24, *p* < .05).

Table 4 presents the results for the tests of Hypothesis 3. Model 1 contained the control variables only. In Model 2, achievement goals and emotion reappraisal were added. In Model 3, the interaction term between achievement goals and emotion reappraisal was added. In support of Hypothesis 3, the interaction effect of achievement goals and emotion reappraisal was statistically significant (*β* = .31, *p* < .01; *R*^2^ = .34, 90% CI [.23, .39]), and there was a significant change in the multiple squared correlation coefficient (Δ*R*^*2*^).

**Table 4:** Results for moderation from hierarchical regression

	Life satisfaction		
	Model 1	Model 2	Model 3
Controls			
Gender	.00	.06	.07
Age	− .02	− .02	− .01
Income	.13	.08	.09
Education	− .03	− .01	.01
Job tenure	.09	.12	.16*
Social desirability	.12	− .02	− .04
Main effects			
Achievement goals		.34**	.35**
Moderators			
Emotion reappraisal		.26**	.23**
Interactions			
Achievement goals × emotion reappraisal			.31**
*F*	1.56	9.07**	12.38**
*R* ^2^	.04	.25	.34
Δ*R*^2^		.21**	.09**
90% CI	[.00, .07]	[.14, .29]	[.23, .39]

CIs are confidence intervals for effect sizes; *N* = 225

^*^p < .05

^**^p < .01

**Fig. 1:**
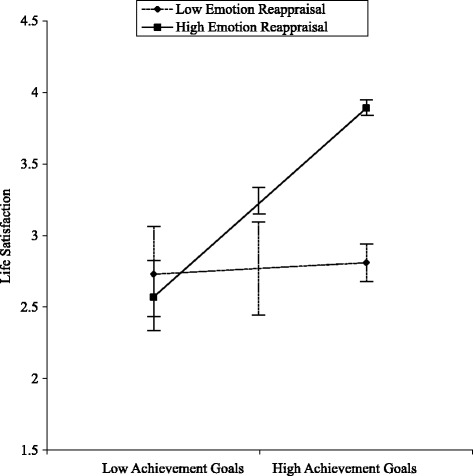
Simple slope analyses. Moderating effect of emotion reappraisal on the relationship between achievement goals and life satisfaction. Error bars represent standard errors

Figure 1 shows that the effect of the two-way interaction between achievement goals and emotion reappraisal was in the expected direction. Following the simple slope analyses, we found that achievement goals at a high level of emotion reappraisal were positively related to life satisfaction (*β* = 1.29, *p* < .01), whereas achievement goals at a low level of emotion reappraisal were not significantly related to life satisfaction (*β* = .10, *p* > .30).

**Fig. 2:**
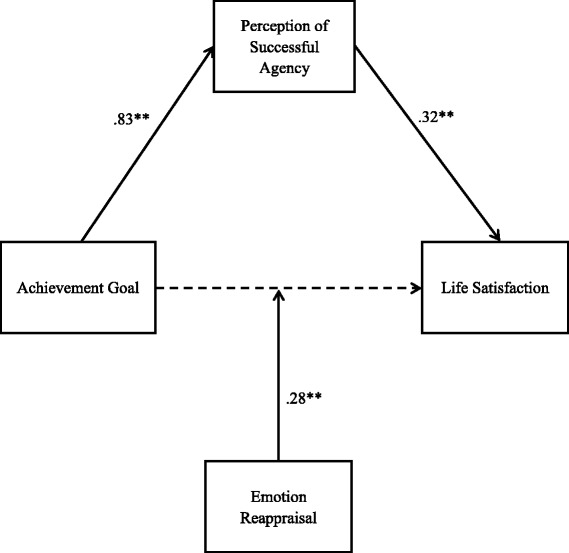
Conceptual and statistical diagram. Research model with important coefficients. Effect size *R*^2^ = .34, 90% CI [.24, .40]

Finally, a model that included both successful agency and emotion reappraisal was tested. Figure 2 illustrates the coefficients (*R*^2^ = .34, 90% CI [.24, .40]). The interaction term remained significant and the indirect effect of achievement goals on life satisfaction through successful agency was also significant (*b* = .27, SE = .09, 95% CI [.10, .44]). These results provided convergent support for our hypotheses.

## Discussion

This study used original survey data to examine the influence of achievement goals on life satisfaction, the mediating role of perception of successful agency, and the moderating role of emotion reappraisal. Consistent with our predictions, achievement goals are positively associated with life satisfaction. Furthermore, we show that this relationship is mediated by perception of successful agency. The simple slope analyses reveal that the positive relationship between achievement goals and life satisfaction holds when emotion reappraisal is high but not when it is low.

The present research contributes to the literature in three ways. First, we contribute to the scant literature on the relationship between achievement goals and life satisfaction. Our findings help fill this gap by showing that achievement goals are positively correlated with life satisfaction. The previous literature provides indirect and mixed evidence for this relationship (Lee et al., 2003; Senko & Harackiewicz, 2005). We reconcile these differences by empirically testing this relationship, thereby adding to the literature investigating the consequences of achievement goals (e.g., Harackiewicz et al., 2000; Cury et al., 2002; Lee et al., 2003; Flanagan et al., 2015). This finding is also in alignment with previous research documenting the overlap between aspiration and well-being (e.g., Cheng et al., 2014; Gao & Smyth, 2010). However, aspiration involves positive expectations regardless of how much effort has been exerted, which obviously should positively contribute to happiness. In contrast, the present research shows that even if considerable effort must be devoted, setting achievement goals is still beneficial to well-being. Therefore, this study complements previous findings by going beyond the aspiration effect.

Second, we identify the psychological process through which achievement goals are related to life satisfaction. Therefore, we shed some light on the role of perception of successful agency in the relationship between achievement goals and life satisfaction. This finding is consistent with the extant literature showing hope as a positive predictor of life satisfaction (Bailey et al., 2007; Bronk et al., 2009; O’Sullivan, 2011). Our research further demonstrates that perception of successful agency, as a dimension of hope, also contributes to life satisfaction. Given that hope is a multidimensional construct and that little research has probed into its sub-dimensions’ downstream effects, the present research serves as a pioneer study.

Third, we examine the moderating role of emotion reappraisal to provide a richer understanding of the relationship between achievement goals and life satisfaction. We show that by cognitively reappraising emotion, people who set achievement goals live a happier life. This result is in line with a body of research that elucidates the positive function of emotion reappraisal in buffering anxiety and enhancing well-being (Feinberg et al., 2012; Gross & John, 2003). Moreover, self-determination theory suggests a positive link between autonomous motivation and well-being (Ryan & Deci, 2000). Through examining the moderating role of emotion reappraisal, we specify the boundary condition under which the positive relationship between achievement goals, a form of autonomous motivation, and well-being ceases to exist. Thus, our study represents an important advancement in self-determination theory.

The findings also offer valuable insights into practice. For example, enhancing employee’s job satisfaction is of vital importance for many organizations. This research implies that organizations can boost employee’s job satisfaction by inspiring their motivation for achievement goals. In addition, we find that if individuals suffer from failure in the process of goal pursuit, they need to reappraise their emotions to restore well-being. Emotion reappraisal can function as a catalyst for well-being when the situation goes against one’s wishes (Gross & John, 2003). The reason why it helps individuals to be less affected by negative events is that emotion reappraisal ccurs early in the emotion-generative process and alters the trajectory of the emotion before the emotional response is generated (Gross, 2002). This has direct practical implications for career development. Consider a scenario in which an ambitious young man aims high in career development and spares no effort at work to get a promotion. However, it turns out that one of his competing colleagues receives the promotion instead, so he fails to achieve his promotion goal at present. Under this circumstance, if he leverages the emotion reappraisal strategy, he may see the competitive situation as an external force that drives him to become better at work, which is actually beneficial to career development in the long run. Consequently, he may feel more positive instead of frustrated or hopeless. This reappraisal would re-motivate him to continue working hard and improving himself until he succeeds.

This research has several limitations that could be solved in future research. First, caution should be exercised before generalizing our results based on Chinese data to Western societies. The meanings of some constructs may be different in China than in Western societies. Although there is no evidence showing the constructs used in the present study contain inconsistent meanings across different cultures, the previous literature indicates that some well-established concepts in Western culture are perceived in another way in China (e.g., Cheung et al., 2001; Wang, 2007). Future studies can directly test whether this difference applies to the study variables in this research. Second, the study is based on cross-sectional data. Therefore, our findings may not imply causality. In future studies, causal inference may be drawn based on experimental data. Meanwhile, caution should be taken for using cross-sectional data to test mediation (Kline, 2015; Maxwell, Cole, & Mitchell, 2011) because cross-sectional analysis can imply the existence of an indirect effect even when the true longitudinal indirect effect is zero. Adopting a longitudinal design in future research would help provide stronger evidence for the process account. Third, the sample size is limited in the present study. Future research can avoid this problem by adopting the sample size estimation approach suggested by Moinester and Gottfried (2014), which should be done before or at an early stage of a study. Fourth, we only identify one moderator in our model. Scholars may investigate other ways through which the relationship between achievement goals and life satisfaction is moderated. For example, similar to emotion reappraisal, psychological resilience refers to the capacity of positive adaptation in adversity (Ong, Bergeman, Bisconti, & Wallace, 2006). According to this definition, it is also a potential moderator between achievement goals and life satisfaction. Fifth, we do not explore the relationships between different types of achievement goals and life satisfaction. Previous research has shown that different types of achievement goals have competing effects on performance (Grant & Dweck, 2003), self-regulation (Lee et al., 2003), and reactions to imperfection (Stoeber, Stoll, Pescheck, & Otto, 2008); therefore, it is essential to further test whether each type of achievement goal has similar or distinct effects on life satisfaction in future studies. Finally, we did not collect participants’ information regarding whether they work in urban or rural environments, which has been shown to be related to well-being (Liang & Wang, 2014). Future research should control for this variable.

## Conclusions

Through a survey study of 225 participants in China, we find that achievement goals are positively related to life satisfaction. Furthermore, the relationship between achievement goals and life satisfaction is mediated by perception of successful agency and moderated by emotion reappraisal. This research provides a comprehensive understanding of how, why, and when achievement goals boost life satisfaction, which is theoretically contributive and practically important.

## Abbreviations

CFI: Comparative fit index
CI: Confidence interval
IPIP: International Personality Item Pool
NNFI: Non-normed fit index
RMSEA: Root mean square error of approximation
SE: Standard error
